# Does intermittent hypoxic exposure enhance the cardioprotective effect of exercise in an inactive population?

**DOI:** 10.3389/fphys.2022.1005113

**Published:** 2022-11-21

**Authors:** Catherine A. Lizamore, Lee Stoner, Yaso Kathiravel, John Elliott, Michael J. Hamlin

**Affiliations:** ^1^ Department of Tourism, Sport and Society, Lincoln University, Christchurch, New Zealand; ^2^ Department of Exercise and Sport Science, University of North Carolina at Chapel Hill, Chapel Hill, NC, United States; ^3^ Department of Medicine, University of Otago, Christchurch, New Zealand

**Keywords:** heart rate variability, heart disease, pulse wave analysis, altitude, cardiovascular disease

## Abstract

The aim of this study was to determine whether exercise supplemented with passive intermittent hypoxic exposure (IHE) improved overall cardiovascular disease risk and individual risk factors. Participants were randomized to exercise-only (Ex, *n* = 18, 5 males, 13 females; age: 56.4 ± 6.5 years; weight: 81.2 ± 15.9; height: 167.3 ± 8.42) or exercise + IHE (IHE + Ex, *n* = 16; 6 males, 10 females; age: 56.7 ± 6.4 years; weight: 78.6 ± 12.4 kg; height: 168.0 ± 8.8 cm). Both groups received the same strength and aerobic exercise training (1 h, 3 days/wk, 10 weeks). IHE + Ex also received IHE (5 min hypoxia: 5 min ambient air ×6) for 2–3 days/wk. Measurements were collected before (Baseline), after (Post), and 4- and 8-week following the intervention. There were small, beneficial reductions in overall 5- year cardiovascular risk in both groups. At Post, for IHE + Ex compared to IHE there were unclear to likely improvements in high density lipoprotein (8.0% ± 8.0%), systolic blood pressure (−3.4% ± 3.4%) and VO_2peak_ (3.1% ± 7.7%). These improvements persisted at 8-week. There was an unclear improvement in arterial wave reflection (augmentation index) at Post (−6.1% ± 18.4%, unclear), but became very likely harmful at 8-week (8-week: 24.8% ± 19.7%). The conflicting findings indicate that in inactive adults, the addition of IHE to exercise may be beneficial to systemic markers of cardiovascular health but may also increase myocardial load due to increased arterial wave reflection.

## 1 Introduction

It has been well established that exercise is a highly effective means of reducing cardiovascular disease and its risk factors (Garber et al., 2011). In an attempt to enhance the health benefits of exercise, some researchers have investigated the use of either exercise supplemented with passive simulated altitude exposure (Balykin et al., 2004) or exercise in a hypoxic environment (Geiser et al., 2001; Friedmann et al., 2003). The motivation behind the inclusion of a hypoxic stimulus to the exercise training is likely related to the adaptations associated with the actions of the alpha subunit of the transcription factor, Hypoxic Inducible Factor—1 (HIF-1α). In hypoxia, HIF-1α acts to stimulate genes responsible for adaptations such as angiogenesis and erythropoiesis (Semenza, 2009), adaptations which are thought to have a beneficial effect on exercise, resulting in greater improvements in exercise capacity than when training in normoxia.

Research regarding exercise training in hypoxia in a sedentary population has returned mixed findings. For example, some studies have reported improved muscle and mitochondrial volume and greater capillary length density (Geiser et al., 2001), improved vascular health (Nishiwaki et al., 2011), and greater VO_2peak_ (Katayama et al., 1998). Other studies have reported either no worthwhile effect of training in hypoxia compared to normoxic training on strength (Friedmann et al., 2003) or mitochondrial function (Pesta et al., 2011). Some research has even reported a detrimental effect on erythrocytes structure and stress tolerance immediately following an exercise intervention in hypoxia (Mao et al., 2011). Others have found that although the hypoxic stimulus resulted in adjustment at the molecular level, this did not transfer into a functional improvement in exercise capacity more so than that of exercise in normoxia (Vogt et al., 2001).

The mix in health outcomes in response to hypoxic exposure may be due to the hypoxic dose. To reduce the physiological stress associated with combined exercise and hypoxic protocols some researchers have investigated the effects of passive hypoxic exposure on health parameters in a sedentary population. To this end, the intermittent, passive hypoxic exposure protocols appear to be most beneficial (Lizamore and Hamlin, 2017), with studies typically demonstrating improved heart rate variability (Lizamore et al., 2016; Chacaroun et al., 2020). However, the lighter dose is also unlikely to yield beneficial changes in body weight or blood pressure (Hobbins et al., 2017).

To our knowledge, there has only been one study which has compared the effect of passive hypoxic exposure as a supplement to normoxic exercise training in a sedentary population. Balykin et al. (2004) examined the effects of an intermittent hypoxic exposure (IHE) technique to simulate altitude in a population of overweight, sedentary males. In this protocol, the participants were seated and passively inhaled 5 min of hypoxic air alternated with 5 min of normoxic air, for 1 h for a total of 10 IHE sessions. The groups receiving IHE in conjunction with 30 min of aerobic exercise on a cycle ergometer at a resistance of 100 W (either immediately following IHE or on alternative days) demonstrated increased parasympathetic and decreased sympathetic activity in the autonomic control of the heart and greater improvements in aerobic capacity compared to either exercise training or IHE on its own. Given the promising results from this study, further research using IHE as a supplement to exercise should be investigated in a sedentary population who may already exhibit autonomic dysfunction and poor cardiovascular fitness.

There are several limitations in the literature investigating the potential health benefits of IHE. For example, most studies have examined the effects of IHE on specific risk factors. While this is useful in that it allows the researcher to pinpoint specific responses as a result of adaptation to the hypoxic stimulus, cardiovascular disease is the result of combination of several risk factors, rather than just one (Dahlöf, 2010). Therefore, when assessing the effects of IHE and exercise on cardiovascular health it is useful to examine the overall cardiovascular profile of the participant in addition to individual risk factors. As sedentary, middle-aged participants have a higher cardiovascular risk than a healthy young cohort, and therefore have more to benefit from such a treatment than a healthy young cohort, more research regarding the effects of IHE on health in an older, inactive population is warranted. Finally, the long-term effects of IHE interventions are seldom measured, and therefore it is unknown how long any potential adaptation associated with hypoxic exposure will last.

Therefore, the purpose of this investigation is to determine the effect of adding IHE to a standard exercise programme on cardiovascular health in sedentary, middle-aged participants.

## 2 Methods

### 2.1 Participants

Inactive (completing less than 30 min of moderate-intensity physical activity five or more days of the week), middle aged (45–70 years) participants were recruited from the local community. All interested candidates underwent a medical screening and were excluded if they had an uncontrolled medical condition (uncontrolled hypertension, hypercholesterolemia *etc.*) were smokers, had any melanoma, cardiovascular disease or were recommended against participation by the assessing medical practitioner or their general practitioner. Participants with a stable medical condition or who took regular medication were included.

**FIGURE 1:**
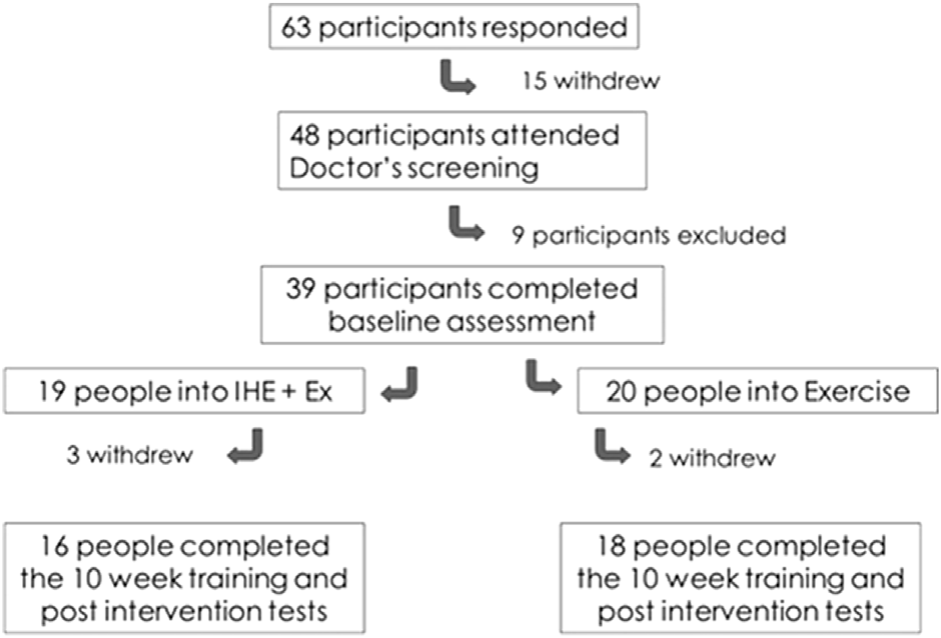
Participant recruitment process.

As outlined in Figure 1, two participants in exercise-only (Ex, *n* = 18) and three participants in intermittent hypoxic exposure and exercise groups (IHE + Ex, *n* = 16) withdrew their participation in the first week of the training program and have been excluded from the group descriptions and analyses. See Table 1 for participant characteristics. All participants were informed of the procedures and risks and provided written informed consent. Human Ethics approval was granted by the Lincoln University Human Ethics Committee (reference 2012-05).

**TABLE 1:** Participant’s baseline data.

	**IHE + Ex (*n* = 16)**		**Ex (*n* = 18)**	
	**Female (*n* = 10)**	**Male (*n* = 6)**	**Female (*n* = 13)**	**Male (*n* = 5)**
Age (years)	56.9 ± 5.8	56.3 ± 7.9	55.4 ± 6.2	59.0 ± 7.3
Weight (kg)	74.0 ± 12.5	86.1 ± 8.5	79.2 ± 17.7	86.5 ± 8.8
Height (cm)	163.6 ± 6.3	175.3 ± 7.5	164.6 ± 8.1	174.2 ± 4.7
Body fat (%)	37.5 ± 7.2	23.6 ± 1.2	39.5 ± 5.8	26.4 ± 4.2
Resting heart rate (beats⋅min^−1^)	65.4 ± 10.0	63.8 ± 8.9	62.0 ± 10.3	58.8 ± 14.4
SBP (mmHg)	124 ± 11.0	126.9 ± 14.1	125.7 ± 17.6	119.1 ± 29.7
DBP (mmHg)	71.1 ± 7.6	73.6 ± 3.7	72.3 ± 8.0	69.0 ± 16.5
TC	5.3 ± 1.1	5.7 ± 0.5	5.9 ± 0.5	5.9 ± 1.1
TC:HDL	3.8 ± 1.3	5.1 ± 1.1	4.3 ± 0.7	5.4 ± 2.1
VO_2peak_ (mL⋅min^−1^⋅kg^−1^)	26.8 ± 6.0	34.8 ± 5.0	25.0 ± 3.9	25.4 ± 7.7

IHE + Ex: Group receiving intermittent hypoxic exposure in addition to exercise during the intervention period. Ex: Group receiving exercise only during the intervention period. SBP, systolic blood pressure; DBP, diastolic blood pressure; TC, total cholesterol; HDL, high density lipoprotein; TC: HDL, total cholesterol to high density lipoprotein ratio; VO_2peak_, peak oxygen uptake during exercise to exhaustion.

### 2.2 Experimental design

Following the baseline testing period (Baseline), the participants were stratified by age and sex and then randomly allocated into one of two groups (Ex, or IHE + Ex) for the 10-week intervention period. The Ex group received three exercise sessions per week, while the IHE + Ex group received 2–3 IHE sessions per week in addition to the three exercise sessions. Participants from both groups trained together.

The post-intervention (Post) testing periods took place within 3 days of the conclusion of the intervention and 4- and 8-week post-intervention measurements were taken to assess the longevity of any changes associated with the intervention.

### 2.3 The 10-week intervention

All participants attended 3, hour-long, moderate-intensity exercise sessions per week. Exercising at a moderate intensity for 150–180 min·wk^−1^ duration is likely to achieve up to 75% of the maximum cardiovascular risk reduction of higher intensities and volumes (Kraus et al., 2019). Therefore, a primarily moderate-intensity exercise regime was selected to avoid injuring participants while still conferring cardiovascular benefit. Exercise sessions included a mix of aerobic and strength training and typically included a 10 min warm-up (65%–70% of HR maximum as assessed during the VO_2peak_ assessment), followed by another 15–20 min of walking or jogging at a participant-elected moderate-vigorous intensity of 70%–80% of maximum HR in Week 1, and thereafter at 75%–80% of maximum HR. (Participants could choose to remain near the “moderate” 70%–76% HR_max_, or the more vigorous 76%–80% HR_max_ range (Garber et al., 2011). Participants then completed 20 min of strength training which targeted major muscle groups. Strength training followed ACSM guidelines (Garber et al., 2011) and included two sets of 10–15 reps of approximately 10 different exercises each targeting different major muscle groups. Participants were advised to select a resistance that allowed them to complete the set, but left their muscles fatigued (moderate—hard intensity). Training sessions utilised free weights and/or resistance bands (bicep and tricep curls, anterior, and lateral raises), and body-weight exercises (lunges, calf raises, sit-ups, back extension exercises, lateral, front and back straight leg raises, and stepping up and down on a step). Where participants had physiological restrictions to prescribed exercises, alternative exercises were provided. The exercise sessions were concluded with 10 min of light aerobic activity and stretching. All exercise sessions were supervised and the participants were encouraged to communicate with the lead researcher regarding any injury, or discomfort experienced in any of the prescribed training exercises or intensities.

In addition to the above training programme, half of the participants completed 2–3 simulated altitude training sessions per week using the IHE protocol of 5 min hypoxia: 5 min normoxia for 1 h. Hypoxic air was delivered through a hand-held face mask (QuadraLite Facemask, Intersurgical, Intermed, Auckland, New Zealand) fitted with an antibacterial filter (HMEF Clear-therm, Intersurgical, Intermed, Auckland, New Zealand). Hypoxic air (Era®-II Hypoxic Air Generator, GO2Altitude, Biomedtech, Victoria, and Australia) was delivered to a main control computer which allowed the researcher to control the fraction of inspired oxygen (F_I_O_2_). Hypoxic dose was monitored using S_p_O_2_ measured at the finger with a pulse oximeter (Nonin Pulse Oximeter, Plymouth, Minnesota). The F_I_O_2_ was progressively lowered to yield the following S_p_O_2_ targets: Week 1: 90%, Week 2: 85%, and Week 3–10: 80%.

#### 2.3.1 Testing periods

**FIGURE 2:**
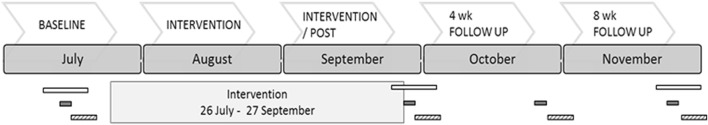
Research timeframe. The bars indicate where various measurements took place, and the interval for measurement. Open bars indicate the period for blood collection, solid bars indicate where arterial stiffness measurements were taken (over 1 week end), and striped bars outline where fitness assessments were measured (over 1 week, immediately following the arterial stiffness assessment.

Each testing period required the participants to attend three separate appointments: a blood sample, an arterial health assessment, and finally an appointment to measure anthropometric, cardiorespiratory fitness, and heart rate variability (HRV). Participants attended sessions at the same time of day for each returning assessment (Figure 2).

#### 2.3.2 Food and activity monitoring

Participants were asked to record all food and medicine ingested, and to describe any physical activity for 2 days prior to measurement sessions. This was to enable a comparison in lifestyle factors that may be responsible for any physiological changes before and after the intervention.

### 2.4 Assessment 1: Overall cardiovascular risk

Cardiovascular risk was assessed using the New Zealand Guidelines Group 5-year cardiovascular risk stratification. In this analysis systolic blood pressure, total cholesterol (TC), high density lipoproteins (HDL), age, diabetes status and sex were used to determine each participant’s overall 5-year cardiovascular risk. Following the risk assessment, participants were divided into four different categories (>2.5%, 2.5%–5%. 5%–10%, 10%–15% chance of cardiovascular disease in the next 5 years).

### 2.5 Traditional cardiovascular risk factors

#### 2.5.1 Blood samples

All blood samples were taken following an overnight fast. Blood was drawn at a local medical facility by a trained phlebotomist, and then analysed at an accredited laboratory (Canterbury Health Laboratories, Christchurch). HDL and TC were analysed using an enzymatic essay performed using the Abbott c8000 analyser (Abbott Laboratories. Abbott Park, Illinois, United States) with Abbott reagents.

#### 2.5.2 Anthropometric data

Participants were assessed for height (Mechanical Stadiometer, Surgical & Medical Products, Mentone, Australia) and body composition (InBody230, Biospace Co. Ltd., Seoul, South Korea), including total body fat percentage and lean muscle mass.

#### 2.5.3 Blood pressure

Resting blood pressure was assessed during the supine rest period of the HRV analysis. Participants rested in a supine position for 16 min. The average blood pressure taken from two automated measurements (Omron, HEM-907XL, Matsuzaka City, Japan) at minutes 10 and 16 were used as the participants’ resting blood pressure.

#### 2.5.4 Heart rate variability

The last 5 min of a 10 min supine rest period was used to capture HRV. The RR interval was detected by a polar transmitter belt (Wearlink W.I.N.D, Polar Electro Oy, Kempele, Finland) and relayed and stored on a HR watch (RS800CX, Polar Electro Oy, Kempele, Finland). Data processing methods have been described in detail by (Lizamore et al., 2016). Parasympathetic activity was measured using the root mean square of successive differences (rMSSD) and the RR interval (Task Force of The European Society of Cardiology and The North American Society of Pacing and Electrophysiology, 1996). The standard deviation of all normal-to-normal beats (SDNN), low frequency (LF) and high frequency (HF) data were also reported to enable greater comparability with other studies.

#### 2.5.5 Cardiovascular fitness

Maximal oxygen uptake was assessed using a Modified Bruce Protocol which increases elevation and/or speed every 3 min. The modified version includes two lighter stages at the start (elevation 0°, speed 2.7 km·h^−1^; followed by elevation 2.8°, speed 2.7 km·h^−1^); thereafter it followed the standard Bruce Protocol. Maximal oxygen consumption was assessed using a metabolic cart (MetaMax^®^ 3B; Cortex Biophysik, Leipzig, Germany) which was calibrated daily. A HR monitor and belt (as used in the HRV assessment) were used to record maximum HR during the fitness assessment. The participant was supervised continuously and the test was stopped immediately if any contra-indications to exercise, as outlined in the American College of Sports Medicine guidelines for exercise testing and prescription, became evident (Thompson et al., 2010). Maximal exertion was achieved when the participant satisfied two or more of the following criteria: HR > 90% of age-predicted HR max (220—age); respiratory exchange ratio of >1.15; a rating of perceived exertion of 19 or 20 on the Borg Scale, or the participant wished to stop due to exhaustion. A 5 min warm down at Stage 1 intensity followed. Participants were supervised until the researcher was satisfied that they had recovered sufficiently. All breath-to-breath data were exported to an excel spreadsheet for analysis. The last 30 s prior to maximal exhaustion were used to determine VO_2peak_ (mL⋅min^−1^⋅kg^−1^). Time to exhaustion was measured as the time taken from the start of Stage 1 to the end of the test (min).

### 2.6 Arterial stiffness and wave reflection

#### 2.6.1 Pulse wave analysis

The augmentation index (AIx) which asses the augmentation of the pressure wave generated during systole was measured automatically using a SphygmoCor device (SphygmoCor, AtCor Medical, Sydney, Australia) and integrated software (SCOR Px 7.1, AtCor Medical, Sydney, Australia). Two measurements were taken on the radial artery following 20 min of supine rest (Stoner et al., 2013). If AIx differed by 4% between the two recordings a third recording was taken, and the average of the closest two measurements were used. Data was only accepted if it fell withing the default quality range (average pulse height: 80 units, pulse height variation: 5%, Diastolic variation: 5%, and quality index: >80%).

#### 2.6.2 Pulse wave velocity

The carotid-radial measurement was used to assess pule wave velocity (PWV). While carotid-femoral pulse wave velocity is the gold standard, detecting a clear and reliable femoral pressure can be difficult in participants with metabolic syndrome or obesity (Laurent et al., 2007), which was a likelihood in our participants. For this reason, and its less invasive nature, the carotid-radial measurement of PWV was used in this study. The pressure waves at the carotid and radial sites were recorded sequentially using a high-fidelity tonometer. At least 2 ½ screens of a clear ECG and tonometry recording was required for each site. The distance covered by the pressure wave was estimated by using a tape measure to measure the distance between the radial artery and the suprasternal notch, and the suprasternal notch and the measurement site at the carotid artery (to the nearest mm). Pulse wave velocity is equal to the distance covered over the mean difference in time (Laurent et al., 2007; Atcor Medical, 2008). Diastolic blood pressure/Mean arterial pressure was used to correct the data.

### 2.7 Statistical analysis

We used a spreadsheet (Hopkins, 2006) to calculate the number of participants required in the study with the smallest worthwhile change in systolic blood pressure being 5 mmHg (Whelton et al., 2018) and the typical error or within-subject standard deviation in systolic blood pressure of 12.5 mmHg (An et al., 1999). Using a type 1 error of 0.5% and a type 2 error of 25% the number of participants in a pre-post parallel groups controlled trial was calculated to be 16 per group. We recruited slightly more than this to account for possible drop-outs during the study.

A mixed modelling procedure (Proc Mixed) was used to analyse the repeated measures (Statistical Analysis System, v. 9.3, SAS Institute, Cary, NC). To reduce the effects of non-uniformity of error, the natural logarithm of each dependent variable was used in the data analysis (Hopkins et al., 2009). Fixed effects were the time points (Baseline, Post, 4-week, 8-week) and the group (IHE + Ex and Ex) and their interaction. Dependent variables included, SBP, VO_2peak_, time to exhaustion, PWV, PWA, HDL, TC, and HDL:TC ratio. Random-effects parameters, or variance components, were also included in the model as covariance components, and were estimated using the restricted maximum likelihood (REML) method. A mixed (including both fixed and variance components) linear model was then fitted to the data which allowed greater statistical inference, that is, resistant to unbalanced data (SAS Institute Inc., 2008).

Effect estimates and *p*-values were then converted to magnitude-based inferential statements using a spreadsheet (Hopkins, 2007). Changes from baseline to Post, 4-week and 8-week in the IHE + Ex group were compared to changes in Ex only using the smallest worthwhile change identified by Cohen (1988), that is, 0.2 multiplied by the between subject SD at baseline. Finally, clinical (all variables except HRV), and mechanistic (HRV) assessments were used to provide magnitude-based decisions (Hopkins, 2020). See Table 2 for an overview of participants excluded from analyses and the motivations for exclusion. The data regarding the New Zealand Guidelines cardiovascular risk assessment chart have been presented in pie charts to represent changes between risk categories at each assessment.

**TABLE 2:** Number of participants excluded from respective analyses.

	IHE + Ex (n)	Ex (n)	Reason for exclusion
Anthropometric data			
Body fat (%)	0	0	—
Muscle mass (kg)	0	0	—
Arterial Health			
Pulse wave velocity (ms)	6	3	Unavailability at the time of recording: PWV & PWA: IHE + Ex: *n* = 4; Ex: *n* = 1
Pulse wave analysis (AIx)	5	3	PWV >2 Inconclusive results: IHE + Ex: *n* = 2; Ex: *n* = 4
			PWA >2 Inconclusive results: IHE + Ex: *n* = 1; Ex: *n* = 5
Heart rate variability			
RR interval (ms)	1	4	RR- Interval & rMSSD: Unclear recording: IHE + Ex: *n* = 1; Ex: *n* = 4
rMSSD (ms)	—	—	—
Cardiovascular fitness			
VO_2peak_ (ml·min^−1^·kg^−1^) &	1	3	Test terminated due to hypertension: IHE + Ex: *n* = 1; Ex: *n* = 2
Time to exhaustion (min)	—	—	—
Blood lipids			
TC, HDL & TC:HDL	1	3	Elected not to have blood samples drawn: IHE + Ex: *n* = 1; Ex: *n* = 3
			Omitted sample at Week 8:Forgot/not available: IHE + Ex: *n* = 3; Ex: *n* = 2
			Started taking lipid lowering medication: IHE + Ex: *n* = 2
Systolic blood pressure			
SBP	1	1	On hypertension medication but BP fluctuated substantially between time points: IHE + Ex: *n* = 1; Ex: *n* = 1
Cardiovascular risk			
NZGG	1	4	Missing either SBP and/or blood sample

IHE + Ex, Group receiving intermittent hypoxic exposure and exercise during the intervention period. Ex, Group receiving exercise only during the intervention period. PWA, pulse wave analysis; PWV, pulse wave velocity; RR, interval: average distance between R-R peaks; rMSSD, root mean square successive difference; VO_2peak_, peak oxygen uptake during exercise to exhaustion; TC, total cholesterol; HDL, high density lipoprotein; TC: HDL, total cholesterol to high density lipoprotein ratio; SBP, systolic blood pressure; NZGG, assessment: 5-year risk assessment according to the New Zealand Guidelines Group.

## 3 Results

Food and activity diaries were examined following the 10-week intervention, and participants did not deviate substantially from their habitual food intake and physical activity.

### 3.1 Baseline data

Baseline data are presented in Table 1.

### 3.2 Cardiovascular risk factors

The Ex only group demonstrated a larger overall decrease in TC, but a lower increase in HDL at Post compared to IHE + Ex. (See Table 3).

**TABLE 3:** Between group (IHE + Ex–Ex) analyses for independent risk factors and overall cardiovascular risk from baseline to post-intervention time points.

	Mean change (%) ±90% confidence interval	Chances (%) of increase/trivial/decrease	Qualitative outcome
**Arterial Health**			
Pulse wave velocity (ms)			
Post—baseline	−0.25 ± 11.2	47/3/50	Unclear
4-week—baseline	−4.2 ± 11.5	26/2/72	Unclear
8-week—baseline	−8.1 ± 12.1	13/1/86	Unclear
Pulse wave analysis (AIx)			
Post—baseline	−6.1 ± 18.4	24/11/65	Unclear
4-week—baseline	11.8 ± 18.4	82/7/11	Likely harmful
8-week—baseline	24.8 ± 19.7	97/1/2	Very likely harmful
**Cardiovascular fitness**			
VO_2peak_ (mL⋅min−1⋅kg−1)			
Post—baseline	3.1 ± 7.7	65/17/18	Unclear
4-week—baseline	9.4 ± 8.0	95/3/2	Very likely beneficial
8-week—baseline	7.9 ± 8.3	90/6/4	Likely beneficial
Time to exhaustion (min)			
Post—baseline	3.8 ± 6.3	81/5/13	Unclear
4-week—baseline	−1.3 ± 6.5	33/8/59	Unclear
8-week—baseline	5.0 ± 6.8	87/4/9	Unclear
**Blood lipids**			
Total cholesterol			
Post—baseline	4.4 ± 6.5	86/2/12	Likely harmful
8-week—baseline	6.5 ± 7.2	93/1/6	Likely harmful
High density lipoprotein			
Post—baseline	8.0 ± 8.0	95/0/5	Likely beneficial
8-week—baseline	10.0 ± 8.5	97/0/3	Very likely beneficial
Total cholesterol: High density lipoprotein ratio			
Post—baseline	−77.2 ± 50.5	1/0/99	Very likely beneficial
8-week—baseline	−24.7 ± 52.4	21/1/78	Unclear
**Systolic blood pressure**			
SBP (mmHg)			
Post—baseline	−3.4 ± 3.4	0/43/57	Possibly beneficial
4-week—baseline	0.5 ± 3.5	12/83/5	Possibly trivial
8-week—baseline	−3.5 ± 3.7	0/42/58	Possibly beneficial

All data are reported as a mean change from baseline in the group receiving exercise and intermittent hypoxic exposure compared to the exercise only group. (A positive outcome indicates a greater relative increase in the group receiving exercise and intermittent hypoxic exposures, compared to the change in the exercise only group). POST, 4-week and 8-week: measurements taken immediately, 4-week and 8-week following the intervention compared to baseline assessments; AIx: Augmentation index; VO_2peak_: peak oxygen uptake during maximal exercise; SBP: systolic blood pressure.

The PWV decreased in both groups following the 10-week intervention (See Table 3). While the IHE + Ex group demonstrated a seemingly lower PWV at the 4-week and 8-week follow up testing period, there were no clear differences between IHE + Ex and Ex.

Immediately following the 10-week intervention there was an unclear decrease in the augmentation index in the IHE + Ex group compared to the Ex. However, in the 4- and 8-week follow up assessments, the augmentation index was reversed (i.e. increased) in the IHE + Ex group compared to Ex group (See Table 3).

The IHE + Ex group showed a small increase in VO_2peak_ at each of the time points (except at the 8-week follow up where VO_2peak_ returned to baseline). Conversely the Ex group remained unchanged between Baseline and Post, increased at 4-week follow up but then decreased to below the baseline value at the 8-week follow up (See Table 3).

### 3.3 Heart rate variability

While rMSSD appeared to increase in the IHE + Ex group more so than Ex immediately post-intervention, this effect was unclear. (See Table 4).

**TABLE 4:** Between group changes (IHE + Ex–Ex) in HRV from baseline to post-intervention time points.

	Mean change (%) ±90% confidence interval	Chances (%) of increase/trivial/decrease	Qualitative outcome
**Heart rate variability**			
RR interval (ms)			
Post—baseline	3.1 ± 5.1	0/100/0	Most likely trivial
4-week—baseline	0.03 ± 5.3	0/100/0	Most likely trivial
8-week—baseline	3.4 ± 5.4	0/100/0	Most likely trivial
rMSSD (ms)			
Post—baseline	18.0 ± 24.0	87/5/8	Unclear
4-week—baseline	14.0 ± 25.2	78/7/15	Unclear
8-week—baseline	13.0 ± 25.6	76/7/17	Unclear
SDNN (ms)			
Post—baseline	1.7 ± 23.1	48/12/39	Unclear
4-week—baseline	−9.9 ± 23.9	20/10/70	Unclear
8-week—baseline	0.9 ± 24.9	46/12/42	Unclear
HF (ms2)			
Post—baseline	52.7 ± 50.0	69/31/0	Possibly increased
4-week—baseline	31.1 ± 52.9	41/57/2	Possibly trivial
8-week—baseline	31.7 ± 53.1	42/56/2	Possibly trivial
LF (ms2)			
Post—baseline	−11.0 ± 51.5	4/81/15	Likely trivial
4-week—baseline	27.9 ± 54.2	31/67/2	Possibly trivial
8-week—baseline	7.4 ± 54.9	14/80/6	Unclear
LF:HF			
Post—baseline	−86.4 ± 56.2	0/1/99	Very likely decreased
4-week—baseline	−15.0 ± 58.3	23/22/55	Unclear
8-week—baseline	1.3 ± 59.6	40/23/37	Unclear

Data are mean change from baseline in IHE + Ex relative to the Ex group (i.e., IHE + Ex–Ex at each time point; thus a positive number indicates a greater value in IHE + Ex compared to Ex) Post, 4-week, 8-week: Measurements taken immediately after, 4- and 8-week following the intervention; All measurement are taken from the last 5 min of a 10 min supine rest interval while participants breathed spontaneously. RR, interval: average distance between R–R peaks; rMSSD, root mean square successive difference; SDNN, standard deviation of normal to normal peaks; HF, high frequency; LF, low frequency; LF: HF, low frequency: high frequency ratio (LF/HF*100).

### 3.4 Body composition

While both groups decreased body fat mass over the course of the 10-week intervention and continued to decrease until the 4-week follow up, the declines in body fat were substantially greater in the Ex group than the IHE + Ex group (see Table 5). Additionally, fat mass in the IHE + Ex group had returned to baseline levels at the 8-week follow up whereas the Ex group continued to decrease. Unlike the changes in fat mass, the IHE + Ex groups demonstrated likely beneficial improvements in muscle mass compared to Ex at Post and at 4-week follow up.

**TABLE 5:** Between group changes (IHE + Ex–Ex) in body fat % and muscle mass from baseline to post-intervention time points.

	Mean change (%) ±90% confidence interval	Chances (%) of increase/trivial/decrease	Qualitative outcome
**Muscle mass (kg)**	—	—	—
Post—baseline	1.4 ± 1.5	56/43/0	Possibly beneficial
4-week—baseline	1.2 ± 1.5	46/53/0	Possibly beneficial
8-week—baseline	0.6 ± 1.5	23/75/2	Unclear
**Fat mass (kg)**	—	—	—
Post—baseline	−0.7 ± 3.9	12/59/29	Unclear
4-week—baseline	2.3 ± 3.9	56/21/3	Possible harmful
8-week—baseline	4.1 ± 4.0	81/18/1	Likely harmful

Data are mean change from baseline in IHE + Ex relative to the Ex group (i.e. IHE + Ex–Ex at each time point; thus a positive number indicates a greater value in IHE + Ex compared to Ex) Post, 4-week, 8-week: Measurements taken immediately after, 4- and 8-week following the intervention.

### 3.5 Overall cardiovascular risk

**FIGURE 3:**
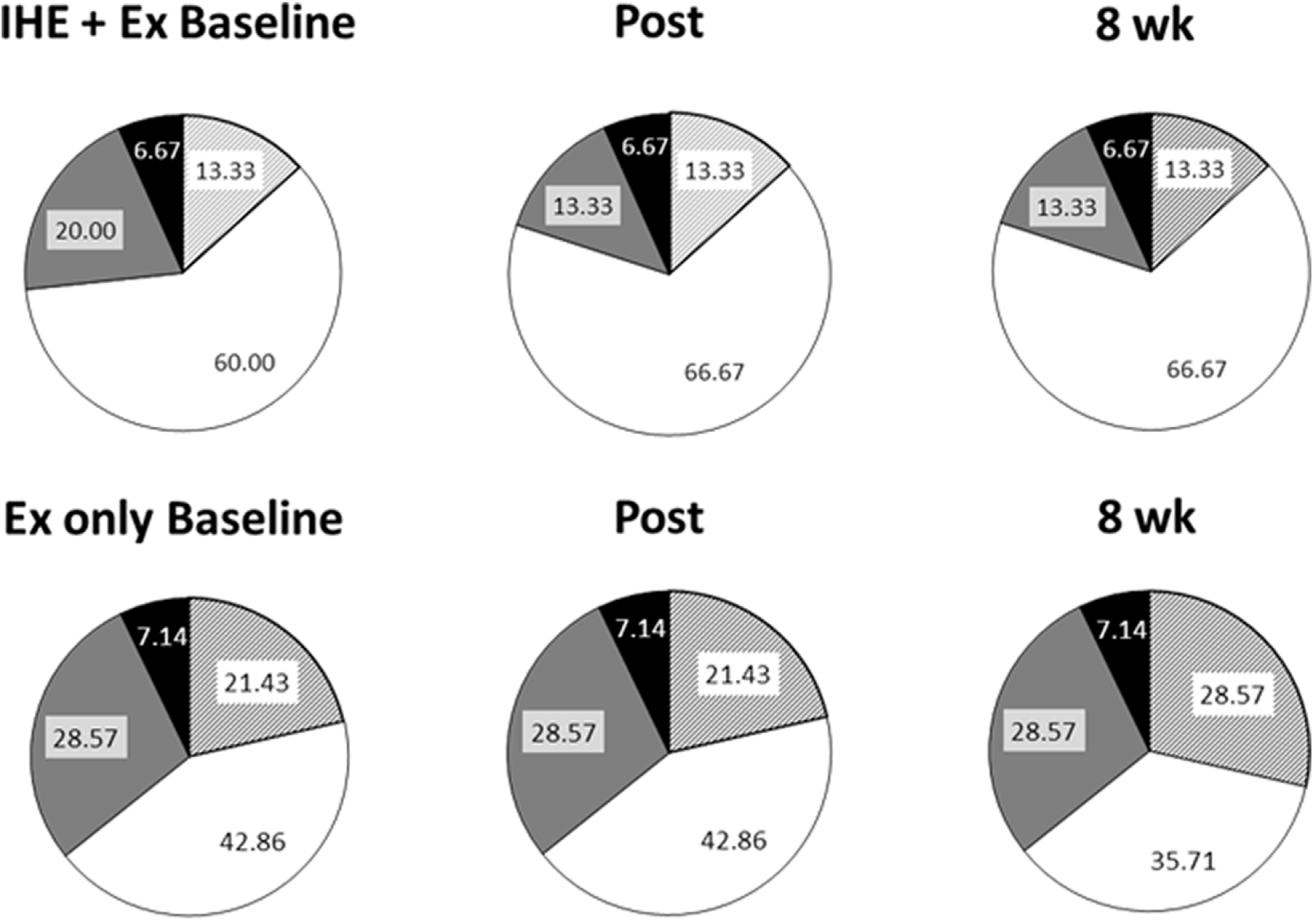
5-Year cardiovascular risk assessed using the New Zealand Guidelines chart. Pie graphs representing the defined risk of cardiovascular disease in the next 5 years as determined by the New Zealand Guidelines Group. Striped portions: <2.5% risk; White portions: 2.5%–5% risk; Grey portions: 5%–10% risk; and Black portions: 10%–15% risk.

Changes in cardiovascular risk are presented in Figure 3. In the IHE + Ex group, there was a small increase in the 2.5%–5% category and a decrease in the 5%–10% category at Post which persisted to the 8-week assessment. In the Ex only group, there were no changes between Baseline and Post, but there was a small increase in the <2.5% risk portion, and a decrease in the 2.5%–5% portion at 8 weeks compared to Post. Based on these observations, it appears that the 10-week intervention had a negligible effect on the most (<2.5% risk) and least (10%–15% risk) healthy participants, but a possibly beneficial effect on those between the 2.5% and 10% risk categories.

## 4 Discussion

The purpose of this study was to assess whether a small number of IHE sessions per week (2–3) over 10-week period would supplement an exercise training programme and yield greater reduction in independent cardiovascular risk factors and overall risk of cardiovascular disease than exercise alone. The key findings from this study are the greater improvements in HDL, SBP and VO_2peak_ following IHE + Ex when compared to exercise only. However, while the augmentation index appeared to decrease slightly (beneficial) compared to Ex immediately post-intervention, there was a likely and very likely increase (harmful) at the 4-week and 8-week follow up. While this could have been due to a decrease in the pulse pressure (attributed to the decrease in SBP in IHE + Ex) relative to the augmentation pressure, more research is needed on the long-term effects of IHE on vascular health before hypoxic-based intervention can be described as “safe”. Contrary to our expectations, there was no clear “additive” effect of IHE on HRV in the participants of this study.

### 4.1 Cardiovascular profile

Changes in the overall cardiovascular risk profiles were subtle. It is likely that below 2.5% risk, there is little more that can be done to improve overall risk (due to non-modifiable risk factors such as age and sex), and at higher risk, perhaps a more aggressive intervention is required before health benefit is observed.

The absence of clearer changes in the overall cardiovascular risk assessment is likely due to the magnitude of the changes needed before a change in risk category is observed. For example, each SBP increment in the New Zealand Guidelines chart spans 20 mmHg, and HDL:TC changes in 1 mmol/L increments. Therefore, the 5-year risk assessment chart is unlikely to have been sensitive enough to detect smaller changes in overall cardiovascular risk as a result of a relatively short intervention.

### 4.2 Blood pressure

The decrease in SBP in our study may be related to several of the other findings in this study, particularly the tendencies for decreased PWV (see Table 3) and increased rMSSD (Table 4). The close relationship between SBP and PWV has long been investigated (Gribbin et al., 1976), with conventional wisdom suggesting that increased SBP is the cause, and the correlated increase in PWV is simply the reflection of the arterial dysfunction associated with the sustained strain on the arterial system (Franklin, 2005). However, several researchers (Dernellis and Panaretou, 2005; Franklin, 2005; Najjar et al., 2008) have challenged this assumption by indicating that the increase in PWV may precede the increase in SBP. The predictive ability of increased PWV in normotensive individuals suggests that PWV is the risk factor, rather than the risk marker. Therefore, the unclear decrease in PWV in the IHE + Ex group in the current study may have contributed to the decrease in SBP in the same group. Alternatively, both the lowered SBP and PWV may be related to the tendency towards an increase in the parasympathetic component (rMSSD), or sympathetic withdrawal in the autonomic nervous system (Palatini and Julius, 2004; Franklin, 2009). That is, both SBP (Guyenet, 2006) and arterial compliance (Boutouyrie et al., 1994; Grassi et al., 1995) are subject to autonomic control. The contribution of sympathetic activity on both the SBP and the PWV has been demonstrated in patients with resistant hypertension who underwent renal sympathetic denervation (Brandt et al., 2012). Following the renal denervation, patients demonstrated substantial reductions in both blood pressure and arterial stiffness. Therefore, the tendency towards improved sympathovagal balance (unclear increase in rMSSD) in the IHE + Ex may also have attributed to the lower SBP in the IHE + Ex group. While the improvements in rMSSD and PWV were unclear, the combined interaction of these variables likely contributed to the clear improvement in SBP in the IHE + Ex group compared to Ex.

However, blood pressure responses to various hypoxic treatments are inconsistent. For example, Hein et al. (2021) noted no statistically significant changes to blood pressure in their older adults following 10 weeks of intermittent hypoxic training. Participants in this study received a set FiO_2_ of 15% which they breathed while they cycled at a heart rate corresponding to 60%, and later 70% of their VO_2peak_. Conversely, other researchers using a passive hypoxic exposure treatment have demonstrated lowered blood pressure results. For example, Panza et al. (2022) observed a statistically significant decrease in systolic blood pressure following 2 weeks of moderate hypoxic intervals of 2 min hypoxia (targeted S_p_O_2_ of 85%–88%) interspersed with 2 min of normoxia. Similarly, Muangritdech et al. (2020) noted significant reductions in blood pressure in the treatment group after alternating 3 min cycles of hypoxic (FiO2 = 0.14) and normoxic (FiO2 = 0.21) bouts for 48 min per day, twice per week for 6 weeks. Therefore, it is likely that both hypoxic severity, type of treatment (during exercise *vs.* passive inhalation), and frequency of treatments will impact the outcomes.

### 4.3 Blood lipids

It is likely that the increase in HDL in the IHE + Ex participants (see Table 3) was responsible for the “possibly harmful” response in TC, and the very likely beneficial response in the TC:HDL ratio. As per blood pressure, changes in blood lipid profile are inconsistent between studies, with some indicating no, or very small changes (Burtscher et al., 2009; Muangritdech et al., 2020) while others report benefit (Tin'kov and Aksenov, 2002). Unfortunately, small sample sizes, lack of control groups, and diverse population groups warrant further examination of the effect of hypoxia on blood lipid profile.

### 4.4 Arterial health

The unclear PWV outcomes in our study may be related to a discrepancy between the training and the testing sites in our experimental design whereby the majority of the exercise was focussed on the lower limbs, while the PWV measurements examined the stiffness of the arteries in the upper-body. Either measuring brachial-ankle PWV, or including more upper body training routines, may have teased out any differences in PWV associated with the IHE + Ex.

The increased augmentation index in the IHE + Ex group (see Table 3) compared to the Ex only group suggests a possible mal-adaption to the hypoxic intervention. These results are intriguing as they appear to conflict with the other measured responses to IHE (such as the decrease in SBP and unclear improvements in rMSSD and PWV in the IHE + Ex compared to Ex).

The increase in augmentation index could be due to a stiffening of the smaller arterioles resulting in a larger intensity of the reflected wave (Kelly et al., 2001). Speculatively, an up-regulation of the endothelial NO synthase pathways during the intervention may have resulted in down-regulation of endothelial NO synthase activity in the post-intervention phase, and subsequently increased arterial vasoconstriction. Kelly et al. (2001) suggests that as the central arteries are predominantly elastic, acute increases in vascular tone may not be readily detected with PWV, but would increase the intensity of the reflected wave and in so doing, increase AIx. As such, while the immediate effects of IHE on arterial stiffness are promising, future research should examine the longevity of changes associated with arterial health (particularly regarding the AIx) that are associated with IHE or hypoxic exercise. In addition, PWV measurements should be tailored to the primary segment involved in the intervention-based training (i.e., carotid-radial in upper-body interventions, and carotid-ankle for lower-body intervention, or carotid-femoral for risk stratification) to get a better indication of overall changes in vascular health.

### 4.5 Heart rate variability

The absence of any clear advantage of IHE + Ex compared to Ex on HRV may be related to the small dosage of hypoxia. As exercise has also been known to increase the RR interval and rMSSD (Jurca et al., 2004; Sandercock G. et al., 2005), the exercise stimulus may have out-weighed any clear additional benefit of IHE to the parasympathetic activity of the nervous system.

It is also important to consider the wide variability associated with HRV, particularly in individuals with compromised health (Sandercock G. R. H. et al., 2005). To this end, reporting a weekly HRV average may be more reliable than a single measurement (Le Meur et al., 2013; Plews et al., 2013). Therefore, future research regarding the use of IHE in an inactive population should consider using the average of a week’s worth of HRV data, rather than a single measurement, in order to elucidate any beneficial (or harmful) effects of IHE on HRV.

### 4.6 Cardiovascular fitness

We had anticipated a larger increase in the VO_2peak_ in both groups as a result of the combined action of the intervention (Murias et al., 2010) and the seasonal change from winter to spring (Ingemann-Hansen and Halkjær-Kristensen, 1982). Additionally, the decrease to below baseline in the 8-week assessment was also surprising and is difficult to account for as any alterations in environmental or testing conditions would have also affected the IHE + Ex group. One would anticipate a decrease in VO_2peak_ following a time interval of more than 2 weeks of inactivity (Neufer, 1989) but this detraining effect is typically halted at approximately baseline values (assuming participants have remained in good health).

The difference between the groups, while small, indicates an additive effect of the IHE on peak oxygen uptake compared to exercise alone. The improvement in VO_2peak_ in the IHE + Ex group could be attributed to haematological adaptation, such as an increase in haemoglobin, acting to increase the oxygen-carrying capacity of the blood, as has been reported following other IHE-based interventions (Burtscher et al., 2004). Therefore, the inclusion of exercise into the current study may have produced sufficient oxidative stress to induce erythropoiesis in the IHE + Ex group. Despite the statistically beneficial outcome of the IHE + Ex on VO_2peak_, there were no clear changes between groups in the time taken to complete the exercise tests. The absence of a clear change in time to complete the test questions the practical benefit of the relative VO_2peak_ increase in the IHE + Ex group.

### 4.7 Fat and muscle mass

The increase in muscle mass, yet lower levels of fat mass loss in the IHE + Ex group are possibly due to oxygen sensors such as HIF-1α which act to reduce the dependency of the body on oxygen-dependent systems such as oxidative phosphorylation following the IHE protocol. This would include a metabolic shift to anaerobic glycolytic pathways that do not require the break-down of fats for energy provision. In addition, the active cellular switch to glycolysis avoids the accumulation of excessive levels of reactive oxygen species that occur under oxidative stress (Semenza, 2009). Perhaps the IHE sessions prompted a shift to glycolytic metabolism, and the associated increases in lactate production facilitated accelerated improvements in buffering capacity and allowed for greater exertion during the strength training portion of the exercise intervention resulting in greater muscle mass in the IHE + Ex group.

### 4.8 Limitations

Despite being inactive, the participants were in generally in good health. Perhaps a greater change in cardiovascular risk factors would have been evident in a population with a higher cardiovascular risk.

The length of a maximal fitness assessment should be < 10 min (Astorino et al., 2004; Yoon et al., 2007), however, given the wide range of fitness abilities, and the fixed protocol of the Modified Bruce Protocol, some participants took considerably longer than this, and therefore the VO_2peak_ results in the current study may be under-estimated for some participants. Further to this, the males in the IHE + Ex group had a considerably higher VO_2peak_ than the males in the Ex only group which may have confounded the overall results. While these discrepancies make it difficult to compare the absolute VO_2peak_ with other studies, the results should still demonstrate changes in fitness in the individual as the protocol was kept consistent between time points.

It would have been interesting to examine any sex differences between the trials. However, greater samples sizes are necessary to ensure adequate power in our results. Similarly, we would require a larger sample size with a greater spread of ages to determine whether or not there is an age effect associated with the treatments.

The F_i_O_2_ was manually adjusted to achieve the desired S_p_O_2_. This means that there would have been slight variations in the F_i_O_2_ between participants, and the S_p_O_2_ may have altered occasionally.

It is also important to note that there were several participants in this study who were unable to complete all measurements at all time points (see Table 2), which may have de-powered our statistical analysis. Some results should therefore be viewed with caution until substantiated by studies with greater subject numbers.

## 5 Conclusions

The inclusion of IHE into a traditional exercise programme resulted in more favourable changes in SBP, VO_2peak_ and HDL compared to exercise alone. While there was some indication of increased resting parasympathetic activity (assessed using rMSSD) in the IHE + Ex group compared to the Ex only group, none of these changes were clear. Additionally, there were no clear differences between the groups in the time taken to complete the maximal exercise test. Importantly, while the immediate arterial response to IHE + Ex seems to improve augmentation index, the longer-term effects of IHE + Ex on arterial stiffness demonstrated an opposite, increase in augmentation index. It is possible that this increased augmentation index was simply the result of a reduction in the pulse pressure without an associated decrease in the augmentation pressure. Finally, the inclusion of IHE into a traditional exercise programme induced small changes to the overall cardiovascular risk profile, and increased VO_2peak_ more so than exercise alone in our inactive middle-aged participants., While the inclusion of IHE into an exercise programme has enhanced many cardioprotective outcomes associated with exercise training alone, further research should test the long-term effects of hypoxic exposure on vascular health before IHE can be considered a safe intervention in an inactive population.

## Data Availability

The raw data supporting the conclusion of this article will be made available by the authors, without undue reservation.
